# Misperceptions and misuse of Bear Brand coffee creamer as infant food: national cross sectional survey of consumers and paediatricians in Laos

**DOI:** 10.1136/bmj.a1379

**Published:** 2008-09-09

**Authors:** Hubert Barennes, Todisoa Andriatahina, Vattanaphone Latthaphasavang, Margot Anderson, Leila M Srour

**Affiliations:** 1 Institut de la Francophonie pour la Médecine Tropicale, BP 9519, Vientiane, Lao Popular Democratic Republic; 2 Health Frontiers, University of Health Sciences, Vientiane

## Abstract

**Objective** To investigate the use of Bear Brand coffee creamer as a food for infants and the impact on consumers of the logo of a cartoon baby bear held by its mother in the breastfeeding position.

**Design** Interviews with paediatricians throughout the country and a national survey of potential consumers regarding their perceptions and use of the Bear Brand coffee creamer.

**Setting** 84 randomised villages in south, central, and northern Laos.

**Participants** 26 Lao paediatricians and 1098 adults in households in a cluster sampling.

**Results** Of the 26 paediatricians, 24 said that parents “often” or “sometimes” fed this product to infants as a substitute for breast milk. In the capital city, paediatricians said that mothers used the product when they returned to work. In the countryside, they reported that poor families used it when the mother was ill or died. Of 1098 adults surveyed, 96% believed that the can contains milk; 46% believed the Bear Brand logo indicates that the product is formulated for feeding to infants or to replace breast milk; 80% had not read the written warning on the can; and over 18% reported giving the product to their infant at a mean age of 4.7 months (95% confidence interval 4.1 to 5.3).

**Conclusion** The Bear Brand coffee creamer is used as a breast milk substitute in Laos. The cartoon logo influences people’s perception of the product that belies the written warning “This product is not to be used as a breast milk substitute.” Use of this logo on coffee creamer is misleading to the local population and places the health of infants at risk.

## Introduction

Food advertising of non-nutritious products targets children throughout the world, not only in developed and wealthy countries.^1^ ^2^ ^3^ Children in Lao Popular Democratic Republic (Laos) are targets of food advertising that contributes to poor nutrition, overweight, and underweight. Markets and school playgrounds are full of attractively labelled, non-nutritious foods such as crisps, fizzy drinks, and sweets.

Of particular concern is the breaching of the international code of marketing of breast milk substitutes, which has been repeatedly advocated in recent years in developing 4 5 and industrialised countries.^6^ The code is intended as a minimum requirement by all governments and aims to protect infant health by preventing inappropriate marketing of breast milk substitutes. “The code applies to the marketing of breast-milk substitutes . . . including bottle-fed complementary foods, when marketed or otherwise represented to be suitable for use as a partial or total replacement of breast milk.”^7^ Product labels are often the only source of information available to consumers regarding the content and uses of a product, and public interest groups have documented multiple instances of formula companies providing misleading information on infant formula labels.^8^

In Laos a popular coffee creamer is marketed with an illustration of a mother bear holding a baby bear in the breastfeeding position (figure). The first listed ingredient is sugar and it contains 3.6% of calories as protein and 27.3% as fat. For infant formulas the recommended content is 7.2-12.0% protein and 40-54% fat.^9^ The coffee creamer is sold in many rural roadside shops. The company uses the same Bear Brand logo on its canned sterilised cows’ milk product and on infant formula products for infants from 6 months. A warning on the can states “This product is not to be used as breast milk substitute” in English, Thai, and Lao. There is also an illustration of a feeding bottle with a cross through it.

**Figure fig1:**
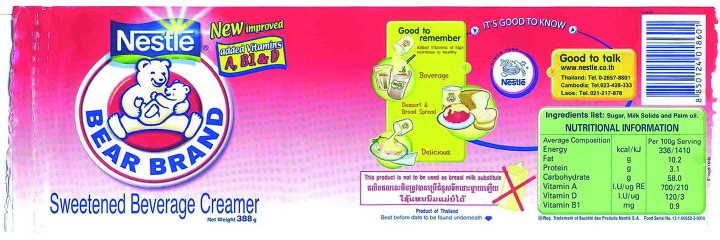
Label from Bear Brand coffee creamer

We have encountered infants and children admitted to hospital with protein-calorie malnutrition who were fed this product exclusively. We conducted two surveys to determine whether or not the practice of feeding this coffee creamer to infants is widespread in Laos. We also explored the impact of the cartoon logo on people’s perception of the product’s appropriateness for infants.

## Methods

### Study site

Laos is one of the poorest countries in southeast Asia, 135th out of 177 countries evaluated according to the human development index.^10^ ^11^ It is a multiethnic and multilingual country with more than 45 languages spoken. The average official literacy rate in Laos (73%) does not reflect the disparity between the rate in urban areas (89%) and that in rural areas (54%), which can be assumed to be overestimated in remote areas.^12^ The documented high prevalence of stunting (41%) and wasting (15%) among children (0-5 years) has not improved over recent decades.^13^ ^14^ Stunting rates reach up to 65% in children aged 12-23 months and in some ethnic groups.^15^ Up to 74% of children aged over 5 in northern rural areas are stunted.^16^ In Laos, 95% of the mothers breast feed their newborn children but the rate of exclusive breast feeding is below 28%.^17^ Food taboos during the postpartum period and inappropriate early supplementary feeding in infants are common nationwide.^18^ ^19^ Inadequate breast feeding and weaning practices contribute to high rates of malnutrition and infant and child mortality.^20^ ^21^ Micronutrient deficiency, beri-beri (clinical vitamin B-1 deficiency), and a high incidence of children with bladder stones have been associated with a diet with low diversity and consumption of glutinous rice.^22^ ^23^

### Study procedure

In Laos in 2006, 26 paediatricians had completed paediatric residency training and were working in eight of the 17 provinces. One author (LMS) interviewed these paediatricians to collect information on parents’ use of the Bear Brand coffee creamer as a substitute for breast milk.

In 2007 we conducted a sample survey in Laos. Of 18 provinces, we chose five as representative of the geographical strata of south (Attapeu, Savannakhet provinces), central (Vientiane Capital and Vientiane provinces), and northern Laos (Luang Nam Tha province). In one district we applied a two stage random sample procedure. At the first stage we randomly selected a village and at the second stage, households. Random numbers were used to select the villages from the list of villages per district and households from the list of households in the villages. We randomly chose one adult per household to be interviewed in the Lao language from among all present household members aged over 18. We randomly selected and enrolled 84 villages and an average of 14 households with one adult from each. A semistructured questionnaire examined knowledge and use of the coffee creamer with the Bear Brand logo.

Participants gave their informed consent to participate in the survey.

### Sample size estimation, definitions, and analysis

We calculated that we needed a sample size of 983 people, based on an estimated use of coffee creamer of 20%, a 4% precision with α=0.05, and 90% power, and we added 10% more people to account for incomplete data, resulting in a sample of 1080.

Data were double entered with Epidata (www.epidata.dk, Odense, Denmark) and Stata, version 8 (Stata Cooperation, College Station, TX). We performed analyses only in people who recognised the coffee creamer can. We used χ^2^ and Fisher’s exact tests for categorical variables and Student’s *t* test and analysis of variance (*F* test) for normally distributed continuous data. We considered P<0.05 as significant.

## Results

Of the 26 paediatricians interviewed, 13 reported that parents “often” feed the Bear Brand coffee creamer to infants as a substitute for breast milk. Eleven reported that parents “sometimes” feed the product to infants. Paediatricians in Vientiane reported that mothers use the product when they return to work. In the countryside poor families use the product as a breast milk substitute when the mother is ill or dies.

Paediatricians have encountered infants and children admitted to hospital with protein calorie malnutrition who had been fed this product exclusively and often reported similar stories (see box).

**Examples of cases**

In the remote countryside of Luang Nam Tha Province an 18 month old boy who weighed 6 kg was admitted to hospital with evidence of kwashiorkor. His grandparents had fed him Bear Brand coffee creamer since the age of 2 months, when his mother had died. The family was of the ethnic group Lenten and did not read the Lao language. They believed the can contained cows’ milk. The child had never grown well and had never moved on to solid foods, but his condition improved rapidly with nutritional support and family education in the hospital.

In the capital city, Vientiane, a 3 month old infant with the oedema and skin changes of kwashiorkor died in hospital from complications of severe malnutrition and pneumonia. Her mother had been concerned that the infant was vomiting with breast feeding shortly after birth. She stopped breast feeding and bought the coffee creamer with the Bear Brand logo. The mother stated that she read the label and understood the creamer was not for use instead of breast milk, but noted that the can had a mother bear with a baby bear on its label, and that people in her village consider this an acceptable infant food.

Table 1 provides the main characteristics of the population surveyed. Of 1098 respondents, 570 (51%) lived in remote rural area, 364 (33%) lived in a semirural area (<30 km from an urban centre), and 164 (15%) lived in an urban area (Vientiane or Savannakhet). Of the adults interviewed, 1031/1098 (94%) recognised the can; 994/1031 (96%) believed that it contained milk; and only 21/1031 (2%) identified the contents correctly as coffee creamer. In total, 191 (19%) reported giving the coffee creamer with the Bear Brand logo to infants at a mean age of 4.7 months (95% confidence interval 4.1 to 5.3). The main reasons given were that they thought it complemented breast feeding (40%), was good for infants’ growth (19%), was a substitute for breast milk (17%), and was cheap (11%).

**Table 1 tbl1:** Characteristics of 1098 adult respondents to survey about use of Bear Brand coffee creamer in Laos

	No (%) or mean (95% CI)
Women	612 (63.6)
Age (years)*	42.0 (41.2 to 42.7)
Illiterate	110 (10.0)
Secondary education or above	559 (50.0)
Ethnic group:	
Lao Loum	1010 (91.9)
Lao Theung	70 (6.4)
Lao Soum	18 (1.6)
Family size*	5.9 (5.7 to 6.0)
Households with no children	132 (12.0)
Households with TV or radio	847 (83.6)
Occupation:	
Farmer	388 (35.1)
Shopkeeper or craftsman	260 (23.6)
No job/housewife/retired	219 (20.0)
Civil servant	129 (11.7)
Worker	33 (3.0)
Other	69 (6.3)

* Mean (95% confidence interval).

Of 1031 people, 824 (80%) said they had not read the text warning on the can. Tables 2 and 3 describe the respondents’ understanding of the Bear Brand logo and the feeding bottle with a cross through it.

**Table 2 tbl2:** Responses of 1023 adults when asked “What does this logo mean?” (respondents shown Bear Brand logo, see figure)

Response	No (%)
Product is good for infants	402 (39.3)
Product is good for children	115 (11.2)
Advertising	110 (10.7)
Product is replacement for breast milk	66 (6.5)
Product is for adults	13 (1.3)
Product is made from animals	6 (0.6)
Bears love their children	4 (0.4)
Product is for everyone	3 (0.3)
Not sure	242 (23.7)
Other response	2 (0.2)

**Table 3 tbl3:** Responses of 1018 adults when asked “What does this picture mean” (respondents shown picture of feeding bottle with cross through it, see figure)

Response	No (%)
Never noticed it/don’t know	557 (49.7)
Product not for infants	319 (31.3)
Product should not be given by bottle	98 (9.6)
Product is dangerous	16 (1.6)
Product is good for babies	10 (1.0)

## Discussion

Bear Brand coffee creamer is a recognised and well distributed product in diverse parts of Laos. Nearly half of surveyed adults believe that the cartoon logo on the can means that the product is “good for infants” or “a replacement for breast milk.” Nearly a fifth of parents had given the product to their young infants. Paediatricians confirmed that parents use this product as a substitute for breast feeding.

Nearly half of respondents did not notice the written warning on the label or the picture of a baby’s bottle with a cross through it. Some (12%) did not understand the meaning of the cross through the bottle. The cartoon bear and her cub seem to provide the most salient misinformation, and the warnings on the label are inadequate to ensure safe and appropriate use of this product. The data suggest that the image of the Bear Brand misleads parents, who believe the coffee creamer is a suitable food for babies.

Protection of breast feeding by limiting the active promotion of the use of breast milk substitutes by formula companies is the central goal of the international code of marketing of breast milk substitutes.^4^ ^24^ ^25^ The sugar based coffee creamer, according to its label, is “not . . . a breast-milk substitute.” The logo on the label of the bear holding a cub in the breastfeeding position, however, conveys quite the opposite message, making this a somewhat different type of code violation.

### Strengths and limitations

Our study was limited by quota samples in four diverse Lao provinces. We interviewed the families at home during the day and therefore interviewed more women. The included villages had to have road access, which excluded more remote villages and limited ethnic diversity. As our sample was more literate than the national average (90% *v* 70%), we might have underestimated the level of misconceptions and the nationwide use of the coffee creamer as a substitute for breast milk because secondary education was associated with a lower risk of giving the coffee creamer to infants (unpublished data).

Formula companies have been shown to provide misleading information on infant formula labels.^8^ We have shown further misconceptions associated with the use of the Bear Brand logo for a coffee creamer. Because of its ease of misinterpretation, this logo should not be permitted on products that are not infant formula.

Our research was limited to Laos, so the relevance to other southeast Asian countries is not known. Future research is required to determine the distribution of this logo in developing countries throughout the world.

### Conclusion

The Bear Brand logo’s non-verbal message implies that the product contained is intended for infants. The powerful visual message is not mitigated by the addition of warning text or by the confusing symbol of the feeding bottle with a cross through it. The sale of coffee creamer with this logo places the health of infants and children at risk in a developing nation that already has extreme levels of malnutrition.

**What is already known on this topic**

Exclusive breast feeding protects infant health and is recommended for the first 6 months of life
The international code on marketing of breast milk substitutes aims to protect infant health by preventing inappropriate promotion of breast milk substitutes and is considered the minimum requirement to be adopted by all governments
Violations have been reported and addressed

**What this study adds**

A cartoon bear holding a cub in the breastfeeding position pictured on cans of coffee creamer implies that the product is suitable for young infants, despite a written warning to the contrary
This product is widely available in the country of Laos and is commonly given to young infants
Use of this logo on coffee creamer is misleading to the local population and places the health of infants at risk

## Notes

We thank the P8 Masters’ students of the Institut de la Francophonie pour la Médecine Tropicale (Agence Universitaire de la Francophonie), J P René, and Bryan Watt; the Lao paediatricians and the staff of Health Frontier, Vientiane; and the families and health authorities of Vientiane Municipality, Savannakhet, Hinheup, Atapeu, and Luang Nam Tha.

Ethical approval: National ethical review board of Laos.

Provenance and peer review: Not commissioned; externally peer reviewed.

## Notes

**Cite this as:** *BMJ* 2008;337:a1379
